# Plumbagin, a Potent Naphthoquinone from *Nepenthes* Plants with Growth Inhibiting and Larvicidal Activities

**DOI:** 10.3390/molecules26040825

**Published:** 2021-02-05

**Authors:** Asifur Rahman-Soad, Alberto Dávila-Lara, Christian Paetz, Axel Mithöfer

**Affiliations:** 1 Research Group Plant Defense Physiology, Max Planck Institute for Chemical Ecology, 07745 Jena, Germany; msoad@ice.mpg.de (A.R.-S.); adavila-lara@ice.mpg.de (A.D.-L.); 2 Research Group Biosynthesis/NMR, Max Planck Institute for Chemical Ecology, 07745 Jena, Germany; cpaetz@ice.mpg.de

**Keywords:** naphthoquinones, plumbagin, *Spodoptera littoralis*, insect growth inhibition, carnivorous plants, *Nepenthes*

## Abstract

Some plant species are less susceptible to herbivore infestation than others. The reason for this is often unknown in detail but is very likely due to an efficient composition of secondary plant metabolites. Strikingly, carnivorous plants of the genus *Nepenthes* show extremely less herbivory both in the field and in green house. In order to identify the basis for the efficient defense against herbivorous insects in *Nepenthes,* we performed bioassays using larvae of the generalist lepidopteran herbivore, *Spodoptera littoralis.* Larvae fed with different tissues from *Nepenthes x ventrata* grew significantly less when feeding on a diet containing leaf tissue compared with pitcher-trap tissue. As dominating metabolite in *Nepenthes* tissues, we identified a naphthoquinone, plumbagin (5-hydroxy-2-methyl-1,4-naphthoquinone). When plumbagin was added at different concentrations to the diet of *S. littoralis* larvae, an EC_50_ value for larval growth inhibition was determined with 226.5 µg g^−1^ diet. To further determine the concentration causing higher larval mortality, sweet potato leaf discs were covered with increasing plumbagin concentrations in no-choice-assays; a higher mortality of the larvae was found beyond 60 µg plumbagin per leaf, corresponding to 750 µg g^−1^. Plant-derived insecticides have long been proposed as alternatives for pest management; plumbagin and derivatives might be such promising environmentally friendly candidates.

## 1. Introduction

**Figure 1:**
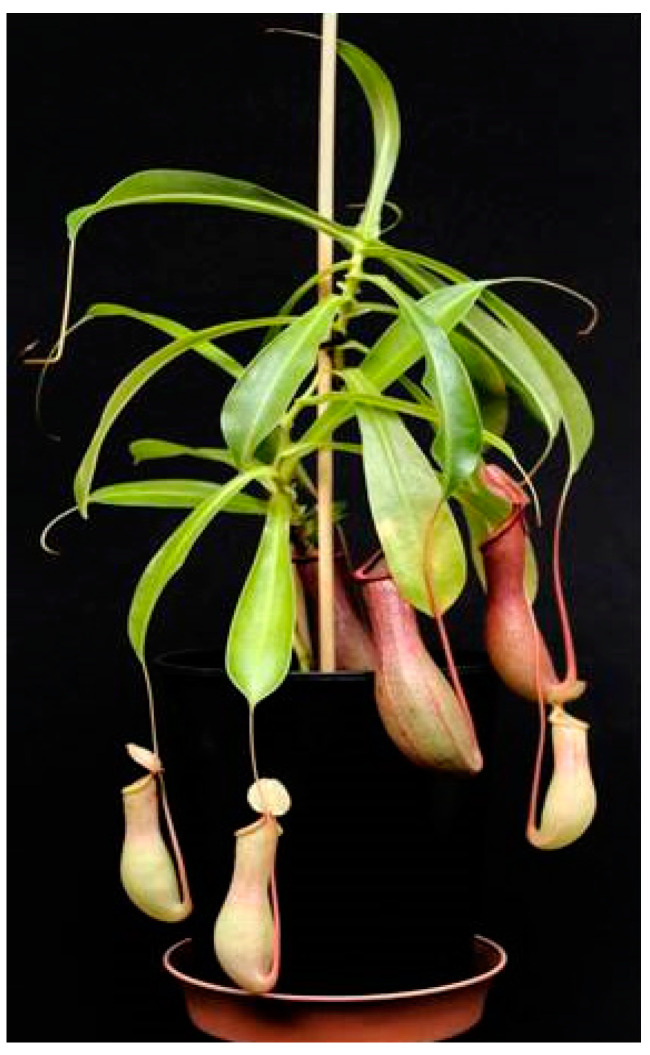
*Nepenthes x ventrata*. A natural hybrid of *N. ventricosa* and *N. alata*. Copyright © A. Rahman-Soad.

*Nepenthes* is a tropical plant genus occurring mainly in Southeast Asia. Plants of this genus are carnivorous. They attract, catch, and digest insect prey in order to get additional nutrients, primarily, nitrogen and phosphate [1,2]. Therefore, *Nepenthes* species developed a pitfall trap (Figure 1), called pitcher, where insect prey falls inside due to a slippery surface and drown in a digestive fluid [1,2]. As in many other carnivorous plants, also the genus *Nepenthes* harbors a large chemical diversity; currently, several secondary metabolites are isolated for pharmaceutical, biotechnological, and ethnobotanical use [3,4]. Especially, *Nepenthes* species are well known in traditional medicine. Multiple reports are in the literature describing curative effects of *Nepenthes* extracts on diseases, e.g., on hypertension, cough, fever, urinary system infections [5], malaria [6,7], pain, asthma [7], *Staphylococcus* infection [8], celiac disease [9], and oral cancer cells [10].

However, up to now, most of the chemical analysis in *Nepenthes* has been done for the digestive pitcher fluid. Here, metabolites with antimicrobial properties have been found, e.g., naphthoquinones (NQ; droserone, 5–*O*–methyl droserone in *N. khasiana* [11]; plumbagin, 7-methyl-juglone in *N. ventricosa* [12]). Thus, it is hypothesized that such compounds mediate protection against microbes and preserve prey during digestion [11,12,13,14]. NQ derivatives are also described for tissues of various *Nepenthes* species including the pitchers [12,15,16,17]. In particular, plumbagin is of broad pharmaceutical interest because it is a candidate that may be used in therapies against various cancers or chronic diseases [18,19,20,21]. In addition to NQ, carotenoids, flavonoids, sterols, and triterpenes are described for *Nepenthes* leaves [1,22,23]. Recently, an untargeted metabolomics approach was performed in *N. x ventrata* comparing secondary metabolites of leaves and pitcher tissue before and after prey catches [24]. In that study, about 2000 compounds (MS/MS events) were detected in the two tissues showing enormous metabolome diversity, which was even higher in leaves. Strikingly, the tissue specificity of chemical compounds could significantly discriminate pitchers from leaves. Besides many yet unknown compounds, the common constituents were phenolics, flavonoids, and NQ [24]. These data suggest that the metabolite composition of the tissues can point to their function. In addition, the metabolite composition may represent mechanisms that promote the evolution of plant carnivory as well as enable the plants to cope with environmental challenges [14].

(A)biotic challenges include the attack of herbivorous insects. Interestingly, there are only a very few observations and studies published concerning the attack of insects on tissues of carnivorous pitcher plants. Recently, lepidopteran herbivory was described for some species of the new world pitcher plant *Sarracenia* [25,26]. There is only one investigation showing that *N. bicalcarata* plants are attacked by an insect, the weevil *Alcidodes spec*. [27]. Another study shows that in *N. gracilis* red pitchers experience less herbivory than green ones [28]. To the best of our knowledge, no other studies have been published yet that focus on herbivore damage in *Nepenthes*. Obviously, the carnivorous syndrome obtained much more attention. However, herbivory on *Nepenthes* tissue is obviously rare. The reason for this is not known but it is unlikely that all herbivores are caught and digested. Instead, *Nepenthes* very likely has an efficient setting of defensive chemistry, which is not unusual in many plants [29]. In order to address this hypothesis and gain more insight in the ecological relevance of *Nepenthes* metabolites, we performed bioassays to study the effect of tissue of *N. x ventrata,* a robust natural hybrid of *N. alata* and *N. ventricosa*, on the feeding behavior and larval development of the generalist insect herbivore *Spodoptera littoralis*.

## 2. Results and Discussion

### 2.1. Effect of Nepenthes x ventrata Tissue on Insect Larvae Growth

**Figure 2:**
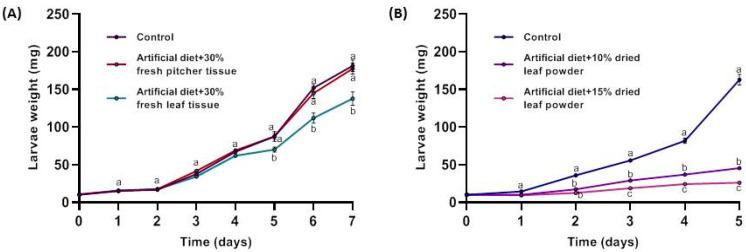
Performance of *Spodoptera littoralis* larvae feeding on artificial diet containing (**A**) fresh leaf powder of *Nepenthes x ventrata* leaf and pitcher (30% (*w*/*w*)) or (**B**) dried *N. x ventrata* leaf powder (10 and 15% (*w*/*w*)) Larvae were weighed every day for 7 days. Mean (± SE) labelled with different letters indicate significant difference (*p* < 0.05); two-way ANOVA, Šidák’s multiple comparisons test; *n* = 15.

The observation that *Nepenthes* plants are rarely infested by insect herbivores forced us to study this phenomenon. Therefore, freshly harvested tissues from *N. x ventrata* leaves and pitchers were added to an artificial diet and fed to larvae of the generalist herbivore *Spodoptera littoralis*. As can be seen in Figure 2A, starting at day 4 to 5, the presence of leaf but not pitcher tissue significantly affected the performance of the larvae, which gained less weight. At this point, it might be worth to mention that recently in *N. x ventrata* [24] and before in *N. khasiana* [15], the concentration of a NQ, very likely plumbagin, was determined to be significantly higher in leaves compared with pitchers, which may explain the result found in Figure 2A. We also could support these results by comparing plumbagin content in pitcher vs. leaf; by quantitative NMR analysis, we found a 5.2-fold higher plumbagin concentration in leaf compared with pitcher tissue (650 and 125 µg g^−1^ FW, respectively). Although significant, the growth inhibition effect was not very pronounced. Thus, the feeding experiment was repeated with dried leaf tissue in order to add more plant material to the diet, knowing that the water content of *N. x ventrata* tissue is about 90% [24]. Here, the effect of the plant tissue was more distinct (Figure 2B). Both quantities of leaf tissue, 10% and 15% (*w*/*w*), showed clear impairment on the growth and weight of the feeding *S. littoralis* larvae already at day 2. Starting from day 3 on, there was also a significant difference between the larvae feeding on either 10% or 15% of *Nepenthes* tissue that was included in the diet (Figure 2B).

### 2.2. Plumbagin in Nepenthes x ventrata Tissue

**Figure 3:**
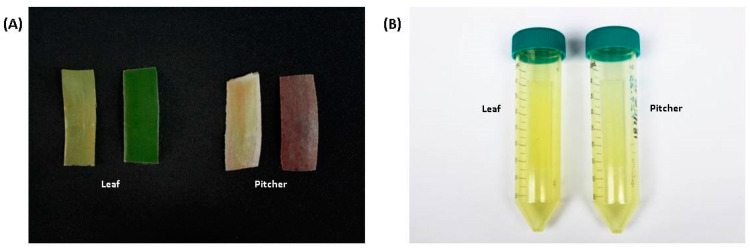
(**A**) Tissues of dry *Nepenthes x ventrata* leaf and pitcher stored for 6 months in a plastic tube. Sublimed compounds cover the dry material with a yellowish color (left) in comparison with freshly cut tissue (right). (**B**) Plastic tubes that stored the different tissue types for 6 months. New tubes do not show any color.

In many carnivorous plants belonging to the order Nepenthales [14], a *sensu stricto* sister group to Caryophyllales [30] and including the plant families Droseraceae and Nepenthaceae, the presence of NQ has been described [31]. This includes species such as *Aldrovanda vesiculosus*, *Dionaea muscipula*, *Drosophyllum lusitanicum,* as well as the genera *Drosera* and *Nepenthes* [31]. Among their secondary compounds, in particular, plumbagin is slightly volatile; thus, its presence in plant tissue is often indicated by spontaneous sublimation, thereby staining the tissue surface or plastic material used for storage. We observed this effect with both leaf and pitcher tissue (Figure 3) stored in plastic vials. In order to proof its identity, a part of the compound was removed from the wall of the plastic vial by extraction with dichloromethane.

**Figure 4:**
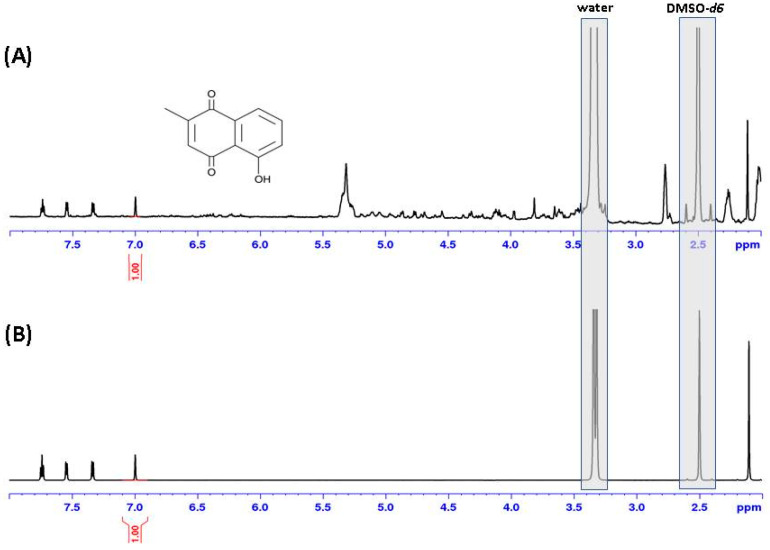

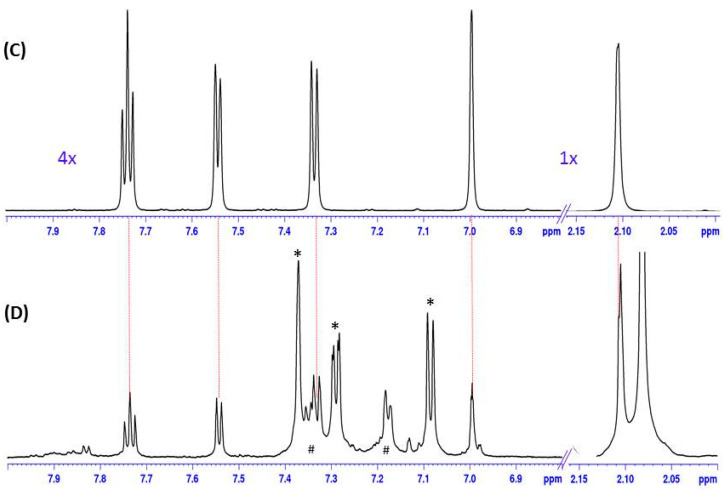
^1^H-NMR spectra in DMSO-*d_6_*. (**A**) Plumbagin (see insert) extracted from *Nepenthes x ventrata* leaves and (**B**) a plumbagin reference. (**C**) Details of ^1^H NMR spectra of a plumbagin reference and (**D**) the volatile exudate emitted by *N. x ventrata* pitcher material. Asterisks (*) indicate the presence of 4-tert-butylcatechol, a polymerization inhibitor probably extracted from the plastic material, and hashes (#) account for an unidentified impurity. The intensity of the aromatic range in (**C**) was increased as indicated by the factor.

After evaporation of the solvent, the residue was used for NMR analysis. In parallel, leaf extracts from *N. x ventrata* were analyzed by ^1^H-NMR as well (Figure 4). When compared with a reference, it could be confirmed that the sublimed volatile compound was indeed plumbagin, and this compound could also be proven in leaf material (Figure 4).

These results raised the question of the function of plumbagin and other NQ in carnivorous plants and in *Nepenthes*. In general, NQ are highly bioactive compounds. Besides pharmacological properties against malaria, various cancers, inflammation, and much more [6,19,32,33,34], they have allelopathic effects as shown for the walnut trees (*Juglans* spp.) releasing the phytotoxin juglone (5-hydroxy-1,4-naphthalenedione) [35,36]. Many defense-related properties are associated with NQ, among them are activities against numerous microbes including human- and phytopathogenic parasites, bacteria, and fungi [31,32,33]. That means, the NQ might protect the plants from pathogen infection. In addition, for *N. khasiana,* it could be shown that droserone and its derivative 5–*O*–droserone provided antimicrobial protection in the pitcher fluid of [11,37]. Buch and coworkers identified plumbagin and 7-methyl-juglone in the pitcher fluid of *N. ventricosa* [12]. These results suggest a role for NQ in the pitcher fluid in order to control the microbiome in the digestive fluid, together with, e.g., pathogenesis-related proteins such as PR-1 [13,37].

### 2.3. Growth-Inhibiting and Larvicidal Activities of Plumbagin

**Figure 5:**
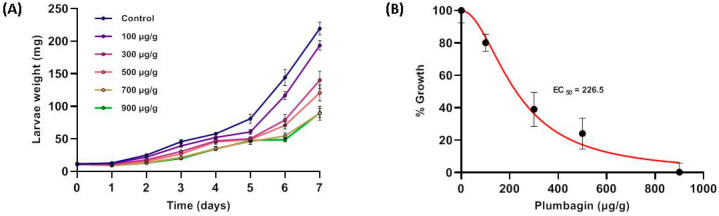
(**A**) Performance of *Spodoptera littoralis* larvae feeding on artificial diet containing various concentrations of plumbagin. Larvae were weighed every day for 7 days. Mean (±SE), *n* = 15. (**B**) Determination of EC_50_ value based on the data obtained in (**A**). EC_50_ was calculated with 226.5 and 1.2 µmol g^−1^ diet, respectively.

Besides the hypothesis that NQ are involved in defense against microbial infection, there are several studies showing that these compounds can also affect insects [31,32,33,38,39,40,41,42,43]. We, therefore, performed feeding experiments with plumbagin-supplemented artificial diet and measured the weight of *S. littoralis* larvae every day. Knowing that the amount of plumbagin in *Nepenthes* leaves is about 0.05% of fresh weight [15], we covered a concentration range between 100 and 900 µg g^−1^, representing 0.01–0.09% fresh mass, respectively. As shown in Figure 5, with increasing plumbagin concentrations, the larvae gained less weight. Based on these data the EC_50_ value was calculated indicating the plumbagin concentration necessary for 50% growth inhibition (weight gain), which was determined as 226.5 µg g^−1^ diet. For some lepidopteran species such as *Spodoptera litura*, *Achaea janata*, and *Trichoplusia ni*, it already has been shown that plumbagin affects the feeding behavior [38,39,40,41]. However, in those experiments, the focus of the analysis was on the level of feeding-avoidance rather than on the larval growth.

**Figure 6:**
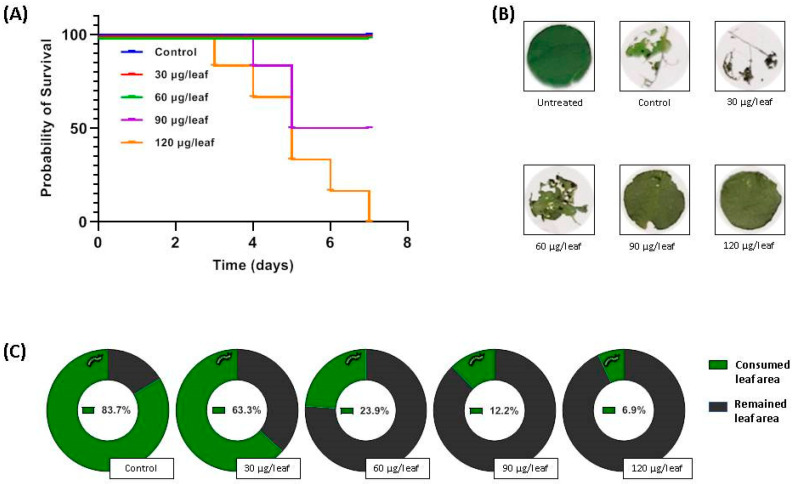
(**A**) Survival rate of *Spodoptera littoralis* larvae feeding on *Ipomoea batatas* (sweet potato) leaf discs painted with various concentrations of plumbagin (*n* = 6). (**B**) Representative leaf discs at the end of the feeding period of day three. Leaf disks were renewed every day. (**C**) Leaf areas consumed by *S. littoralis* larvae (indicated in green) at day 3 depending on the applied plumbagin concentration.

In contrast to most other bioassays that analyzed the antifeeding activity of plumbagin, here, the compound of interest was included in the food, not painted on leaves of various plant species. Nevertheless, in order to determine the mortality rate of larvae feeding on plumbagin, we also carried out an experiment using the approach with plumbagin-painted leaves. Therefore, a sweet potato cultivar (Tainong 66) that is known to be susceptible to herbivores and does not induce strong defense response upon attack was selected [44]. In first experiments, we observed that *S. littoralis* larvae even preferred cannibalism than feeding on those leaves. As a consequence, only individualized larvae were used. Up to a plumbagin concentration of 60 µg^−1^ leaf (13.3 µg cm^−2^, 750 µg g^−1^ leaf) no larvicidal effect was determined for the period analyzed (Figure 6A). With 90 µg^−1^ leaf (20 µg cm^−2^; 1.125 mg g^−1^ leaf) dead larvae could be found at the end of day 4 and the survival rate drop to 50% at the end of day 5. At 120 µg^−1^ leaf (26.7 µg cm^−2^; 1.5 mg g^−1^ leaf), dead larvae were detected at day 3 and until the end of day 7, all larvae have died (Figure 6A). For *T. ni* feeding on plumbagin-covered cabbage leaves, an antifeeding effect was also determined in the low microgram per square centimeter range [41]. It also can be seen that the larvae avoided feeding on the leaves covered with high concentrations of plumbagin (Figure 6B,C). With respect to the results shown in Figure 5, it seems that larval growth is heavily affected at higher plumbagin concentrations of around 700 µg plumbagin g^−1^ diet. However, the larvae were affected in growth but still survived at all concentrations tested (up to 900 µg g^−1^). The plumbagin concentrations used in the no-choice assay also showed no mortality up to 750 µg g^−1^ leaf tissue. Only at the used concentration of 1.125 µg g^−1^ leaf, we found the first larvae dying. This suggests that there might be a threshold of about 1 mg g^−1^ food before the *S. littoralis* larvae begin to die. The experiment is somehow comparable with a recent study by Hu and colleagues [42]. They investigated the mortality of *Pieris rapae* and *Helicoverpa armigera* feeding on cabbage leaves dipped into solutions with different concentrations of plumbagin and juglone, respectively. For plumbagin, IC_50_ values of 11 µg mL^−1^ (*P. rapae*) and 30 µg mL^−1^ (*H. armigera*) were calculated [42]. However, these data are hard to rank as it is not known how much of the compounds of interest was finally on or in the leaf disc. Nevertheless, for all the latter assays, it is difficult to discriminate whether the larvae really die either because of the ingested compounds or of hunger as they consequently avoid feeding. Other studies used topical assays where the compound was added directly onto the insect’s (e.g., *S. litura*, *A. janata*, and *Musa domestica*) body to investigate the toxicity of compounds [38,43]. This approach is worth to carry out but not qualified for studies on activities of compounds that are incorporated during herbivory.

However, the mode of action of NQ is not completely known. In general, NQ are redox-active compounds that can generate oxidative stress [33]; moreover, there are hints for specific inhibition of enzymes and, hence, processes involved in insect development mainly the molting process in insects, e.g., the enzymes phenoloxidase [30], chitin synthetase [45], or ecdysone 20-monooxygenase [46]. The interaction with molting hormone pools is discussed as well [47]. Another study showed that in *Anopheles stephensi,* the level of certain enzymes such as esterases and SOD was decreased significantly in the presence of plumbagin, which also was active as repellent against *A. stephensi* at a concentration of 100 µg mL^−1^. Further histological investigations showed that muscles, midgut, and hindgut were the most affected tissues [48]. However, most studies suggest that, most likely, the insecticidal activity of plumbagin is based on the inhibition of ecdysis. This also includes a certain specificity against insects compared with neurotoxic insecticidal compounds.

Botanical or plant-derived insecticides have long been touted as environmentally friendly alternatives to synthetic insecticides for pest and disease management [3]; NQ combine the advantage of both low toxicity, compared with conventional pesticides, and restricted environmental contamination and, thus, might be promising candidates for an ecological agriculture.

## 3. Materials and Methods

### 3.1. Insects and Plants

*Spodoptera littoralis* Boisd. (Lepidoptera: Noctuidae) were hatched from eggs kindly provided by Syngenta Crop Protection (Stein, Switzerland) and reared on artificial diet (500 g hackled beans, 9 g ascorbic acid, 9 g 4-ethylbenzoic acid, 9 g vitamin E Mazola oil mixture (7.1%), 4 mL formaldehyde, 1.2 L water, 1 g-sitosterol, 1 g leucine, 10 g AIN-76 vitamin mixture, and 200 mL (7.5%) agar-water solution) at 23–25 °C with a 14 h photoperiod. Sweet potato (*Ipomoea batatas* Lam. cv Tainong 66) scions were grown as described [40] under a 16/8 h light/dark regime at 28/25 °C, respectively, and 70% relative humidity. *Nepenthes x ventrata* (*N. alata x N. ventricosa* hybrid) plants were grown at 21–23 °C, 50–60% relative humidity, and a 16/8 h light/dark photoperiod. Pitcher and the associated leaf tissues were harvested at the time when the pitchers were just opened, directly frozen in liquid nitrogen and ground with mortar and pestle. Material was used directly (fresh) or freeze-dried before use.

### 3.2. Feeding Assays

For feeding assays, second to third instar larvae of *S. littoralis* were used. Ground fresh or dried plant material (leaves and pitcher) from *N. x ventrata* was added to the artificial diet with the indicated quantities (*w*/*w*). Plumbagin (5-hydroxy-2-methyl-1,4-naphthoquinone, C_11_H_8_O_3_; Fischer Scientific, Schwerte, Germany) was dissolved in acetone and added to the diet. Controls were prepared in the same way without plumbagin. At all the time, it was made sure that acetone was evaporated. For these feeding assays, 15 independent repeats were done. No-choice leaf disks feeding assays according to [34] were further performed on sweet potato. Therefore, leaf discs of 24 mm in diameter were punched out with a cork borer put directly on wet filter paper in a petri dish (5.5 cm diameter). Plumbagin was solved as described before and diluted to the required concentration with 2.5% (*w*/*v*) PEG 2000 (Sigma-Aldrich, Taufkirchen, Germany). That solution was added onto the surface of the discs at the concentrations indicated. For the no-choice assays, 6 independent repeats were performed.

Every day fresh diet or leaf discs were provided. All assays were performed with individual larvae to avoid cannibalism. Larvae were reared for the indicated periods on the particular diets and weighed at the given time.

### 3.3. Isolation of Plumbagin from Nepenthes x ventrata Leaves

Freshly harvested *N. x ventrata* leaves (7.3 g) were immediately frozen in liquid N_2_ and freeze-dried. Dried tissue was ground and extracted with 100 mL dichloromethane (DCM) for 15 min by stirring in Erlenmeyer flasks. After precipitation for 20 min, the clear supernatant (50 mL) was collected and another 50 mL DCM was added to the remaining material for re-extraction, which was repeated six times. Collected supernatants were filtered, combined, and DCM was removed using a rotary evaporator. The dried extract (9.3 mg) was dissolved in 2 mL DCM transferred into a HPLC vial and dried again under N_2_ stream. For the whole procedure, only glassware was used. The NQ in the extract was identified by means of NMR spectroscopy by comparing spectral data with those of an authentic standard (plumbagin).

*N. x ventrata* leaf material was kept in 50 mL polypropylene tubes at room temperature over 6 months during which the NQ sublimed (Figure 4), leaving a yellowish stained plastic material. Absorbed compounds were extracted from closed tubes with DCM (10 mL) for 3 days at room temperature. The extract was transferred into a glass vial and evaporated using N_2_ gas. The residue was reconstituted with DMSO-*d*_6_ and subjected to NMR analysis.

Identity of the sublimed and extracted plumbagin was confirmed by ^1^H-NMR spectroscopy. NMR spectra were measured on a Bruker Avance III HD spectrometer (Bruker BioSpin GmbH, Rheinstetten, Germany) equipped with a cryoplatform and a TCI 1.7 mm Micro-CryoProbe. Spectra were referenced to the residual solvent signal for DMSO-*d*_6_ at δH 2.50. Spectrometer control and data processing was accomplished using Bruker TopSpin 3.6.1, and standard pulse programs as implemented in Bruker TopSpin 3.6.1 were used.

For a quantitative comparison of ^1^H NMR spectra of extracts of *N. x ventrata* leaf and pitcher tissue, the spectral intensity was adjusted to equal solvent signal areas. The areas of signals accounting for plumbagin (range: δH 8.00–7.00) were determined and used for calculation based on the respective areas of a plumbagin standard. For preparation of the experiment, 729 mg (FW) of each tissue was ground in liquid N_2_ and extracted with 20 mL of dichloromethane in closed vessels at room temperature with shaking. Extracts were filtered through Chromabond PTS phase separation cartridges (Macherey-Nagel, Düren, Germany) and the flow-through was evaporated with N_2_ gas at room temperature within 30 min. Afterwards, the residue was reconstituted with 1.2 mL DMSO-*d*6 and subjected to ^1^H-NMR spectroscopy.

### 3.4. Statistical Analysis

Statistical calculations were performed using GraphPad Prism version 9.0.0 in all cases. Details are indicated in the particular figure legends. For EC_50_ analysis, the total response was normalized to run between 0% and 100% using control data. For growth experiments, larvae were picked randomly from a large population and all experiments were conducted out under highly standardized conditions to avoid investigator-included bias.

## 4. Conclusions

Naphthoquinones are known metabolites in several plant species. Among these are various carnivorous plants including the pitcher plant *Nepenthes*. Plumbagin is a prominent NQ in *Nepenthes x ventrata* and it was detected by ^1^H-NMR in tissues in different concentrations (100 and 650 µg g^−1^ fresh weight in pitcher and leaf, respectively). Plumbagin has known antimicrobial activities and is of pharmaceutical interest. Now, in different feeding assays with *Spodoptera littoralis* larvae the anti-feeding, growth-inhibiting and larvicidal activity of plumbagin or plumbagin-containing tissues was demonstrated at naturally occurring concentrations. Plumbagin as well as other NQ might become alternative compounds as natural insecticides in agriculture.

## Data Availability

All data generated or analyzed during this study are included in the main text.
